# The influence of three acid modifications on the physicochemical characteristics of tea-waste biochar pyrolyzed at different temperatures: a comparative study†

**DOI:** 10.1039/c9ra02729g

**Published:** 2019-06-04

**Authors:** Chathuri Peiris, Oshani Nayanathara, Chanaka M. Navarathna, Yohan Jayawardhana, Samadhi Nawalage, Griffin Burk, Akila G. Karunanayake, Sunith B. Madduri, Meththika Vithanage, M. N. Kaumal, Todd E. Mlsna, El Barbary Hassan, Sachith Abeysundara, Felio Ferez, Sameera R. Gunatilake

**Affiliations:** College of Chemical Sciences, Institute of Chemistry Ceylon Rajagiriya CO 10107 Sri Lanka ranmal@ichemc.edu.lk; Department of Chemistry, University of Colombo CO 00300 Sri Lanka; Department of Chemistry, Mississippi State University Mississippi State MS 39762 USA; National Institute of Fundamental Studies Hantana KY 20022 Sri Lanka; Biochar Supreme LLC Everson WA 98247 USA; Department of Sustainable Bioproducts, Mississippi State University Mississippi State MS 39762 USA; Ecosphere Resilience Research Center, Faculty of Applied Sciences, University of Sri Jayewardenepura Nugegoda CO 10250 Sri Lanka; Department of Statistics and Computer Science, University of Peradeniya Peradeniya KY 20400 Sri Lanka; Material Science Lab, Integrated Microscopy Center, University of Memphis Memphis TN 38152 USA

## Abstract

Tea-waste is an abundant feedstock for producing biochar (BC) which is considered to be a cost effective carbonaceous adsorbent useful for water remediation and soil amendment purposes. In the present study, tea-waste BC (TWBC) produced at three different temperatures were subjected to nitric, sulfuric and hydrochloric acid modifications (abbreviated as NM, SM and HM respectively). Characteristics of the raw and modified BC such as ultimate and proximate analyses, surface morphology, surface acidity and functionality, point of zero charge, cation exchange capacity (CEC) and thermal stability were compared to evaluate the influence of pyrolysis temperature and of modifications incorporated. The amount of carboxylic and phenolic surface functionalities on TWBC was seen to decrease by 93.44% and 81.06% respectively when the pyrolysis temperature was increased from 300 to 700 °C. Additionally, the yield of BC was seen to decrease by 46% upon the latter temperature increment. The elemental analysis results provided justification for high-temperature BC being more hydrophobic as was observed by the 61% increase in H/C ratio which is an indication of augmented aromatization. The CEC was the highest for the low-temperature BC and was seen to further increase by NM which is attributed to the 81.89% increase in carboxylic functionalities. The surface area was seen to significantly increase for BC700 upon NM (∼27 times). The SM led to pore wall destruction which was observed in scanning electron microscopy images. Findings would enable the rational use of these particular modifications in relevant remediation and soil amendment applications.

## Abstract 2

Tea-waste biochar produced under different pyrolysis temperatures were subjected to three acid modifications and a comparative evaluation of their key physicochemical characteristics made.

## Graphical abstract

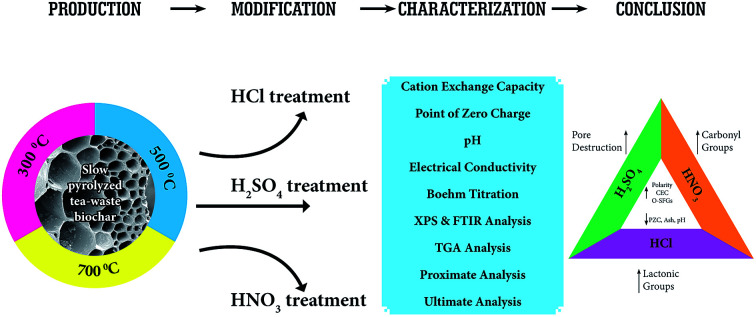

## 1. Introduction

Biochar (BC) is a ubiquitous carbonaceous adsorbent that is gaining colossal research interest worldwide.^1^ Two major commercial applications of BC can be considered as water remediation and agricultural soil amendment.^2–6^ Production of BC helps to control environmental pollution by facilitating solid waste management of biomass, while its carbon negative process has been suggested as a means to combat climate change.^7^ It has rapidly gained attention in recent years due to its cost effectiveness and excellent adsorption properties.^8,9^ Selection of the feedstock is a critical factor in BC production as the feedstock should be inexpensive and readily available for large scale industrial applications.^1,8^

Numerous physical and chemical properties govern the sorption efficiency of a BC. Physical characterizations include surface morphology, surface area, pore size, ash and moisture content whereas factors such as surface functionality, surface acidity, point of zero charge (PZC) and cation exchange capacity (CEC) *etc.* can be determined chemically.^4,10^ These characteristics mainly depend on the feedstock type and pyrolysis conditions.^11^ Higher efficiencies can be obtained by selecting the correct pyrolysis temperature as well as by providing the optimum sorption conditions. Thus, a proper understanding of the BC of interest becomes essential for it to be used in relevant applications.^8^

The CEC of BC is a vital factor that governs the ability of an adsorbent to retain positively charged ions on its surface.^12^ Properties such as specific surface area, elemental composition and the working pH of the medium can be considered as aspects that influence the CEC.^13,14^ The presence of oxygenated surface functional groups (O-SFGs) affect retention capacity significantly as well whilst contributing to the sorption mechanism. These acidic O-SFGs on the BC surface are of three types based on acid dissociation constants; stronger acids (carboxylics; p*K*_a_ ∼ 5–6.4), moderate acids (low p*K*_a_ phenols and the hydrolysis products of lactones; p*K*_a_ ∼ 6.4–10.3) and weaker acids (high p*K*_a_ phenols; p*K*_a_ ∼ 10.3–13).^13,15^ Yet another feature that defines BC functioning is PZC, a property that describes the pH at which the BC surface holds a zero net electric charge.^16^ When in a medium that has a pH higher than the PZC (pH_medium_ > pH_PZC_), the BC surface is said to possess a predominance of negative charge and *vice versa*.^17^ The PZC gives an idea as to whether the surface functional groups (SFGs) are in their protonated or deprotonated form.

Far from the latter, for adsorption *via* pore filling, it is necessary that the pore size of the BC is compatible with the size of the adsorbate. It has been reported that the sorption within pores occur only when the pore size is at least 1.7 times larger than the second widest dimension of the adsorbate of interest.^8^

Conducting an acid treatment influences the physicochemical properties of BC significantly as it can affect both surface functionality and the porous structure of the BC.^18,19^ The pore wall destruction that results as a consequence of acid modification facilitates the pore filling mechanism for different adsorbates.^20,21^ There is also an increase in specific surface area and total pore volume due to demineralization in addition to the increase in surface heterogeneity.^22^ Furthermore, the surface oxidation of the carbonaceous surface introduces new O-SFGs whilst oxidizing existing functional groups.^23^

Nitric acid modification (NM) causes functional group fixation *via* nitration and oxidation, which introduces nitrogen moieties on BC surfaces *via* electrophilic substitution.^22,24,25^ Sulfuric acid modification (SM) converts micropores to mesopores and macropores through pore wall destruction, while also acting as a strong oxidizing agent.^20^ Hydrochloric acid modification (HM) on the other hand causes enhancement of O-SFGs *via* the reduction of carbonyl groups such as lactones, phenols and ethers.^24^

Tea is one of the largest agricultural exports in many tropical and sub-tropical countries including China, Sri Lanka, Kenya, India, Indonesia, Vietnam *etc.*^26,27^ The world annual tea production was as high as 5.73 million tons in 2016^28^ and it reportedly lead to the accumulation of large amounts of waste.^29^ For example, in Turkey, the rejected tea amounted to about 18% of total production.^30^ The effective management of this solid waste becomes important. Therefore, tea-waste is an abundant and low-cost feedstock for BC production in these countries.^30,31^ Tea leaves are porous material having a network like structure.^31^ Biochar produced from tea-waste contains ∼30% holocellulose, ∼25% lignin, and ∼15% extractives.^32^ Tea-waste biochar (TWBC) has become a popular sorbent during the past decade due to the presence of these constituents that add functionalities which enhances the sorptive properties of the BC.^30–32^

To the extent of our knowledge, there has been no extensive study carried out to establish a comparison for the influence in physicochemical properties that take place upon the incorporation of common acid modifications. The presented study is an attempt to fill this research gap by conducting a thorough analysis of characteristics related to both the raw and modified BC produced under three pyrolysis temperatures.

The precursor BC types produced at 300, 500, and 700 °C were subjected to NM, SM and HM and the variations in the surface morphology, acidity and functionality, proximate and ultimate analyses parameters, PZC, CEC and thermal stability observed. Additionally, statistical analyses were carried out and the correlation between parameters were evaluated. The overall results obtained would be a comprehensive evaluation on the effect of different acid modifications on TWBC that would become helpful for the judicious selection of an appropriate adsorbate to be used in applications of interest.

## 2. Material and methods

### 2.1. Reagents, apparatus and equipment

Analytical grade reagents, hydrochloric acid, nitric acid, sulfuric acid, sodium hydroxide pellets, sodium carbonate, sodium bicarbonate, sodium chloride, sodium acetate, ammonium acetate and ethanol were purchased from Sigma Aldrich (St. Louis, MO). Calibration standards for the atomic absorption spectrometer (AAS) were purchased from Inorganic Ventures (Christiansburg, VA). A Nabertherm GmbH, Germany muffle furnace equipped with a hardening box was used for the BC production. A KEM EBU-610-20B automatic titrator, Kyoto Electronics Manufacturing Co. Ltd. (Japan) was used for Boehm titrations. A Metrohm 7025M (Switzerland) digital pH meter and a Thermo Scientific, (Waltham, MA) model 5 STAR electrical conductivity meter was used for pH and conductivity measurements. A Daihan Scientific, (Korea) water bath shaker was used for drying purposes. A Hitachi ZA3000 polarized Zeeman AAS (Japan) was utilized for the determination of CEC. For the functional group analysis, ABB MB300D Fourier transform infrared spectrophotometer (Switzerland) with attenuated total reflection (ATR) probe was incorporated. Total carbon (C), nitrogen (N), hydrogen (H) and oxygen (O) content of BC samples were determined by dry combustion, using a CE – 440 (Exeter Analytical INC.) elemental analyzer. X-Ray Photoelectron Spectroscopy (XPS) analyses were performed using a Thermo Scientific K-Alpha (K_α_) XPS system equipped with a monochromatic X-ray source. Thermal stability of BC were determined using a thermogravimetric analyzer (TGA) (TA instruments, TGA Q 50). Scanning electron microscopic (SEM) images were obtained by a JEOL JSM-6500F FESEM instrument at 5 kV. Surface area analysis was carried out using a Micromeritics' TriStar II PLUS surface area and porosity system.

### 2.2. Biochar production

Tea-wastes were collected from three factories in Sri Lanka located in Nuwara eliya, Matale and Ambalangoda belonging to the subtropical highland, intermediate and tropical climate zones respectively. Dried biomass (length: ∼3.87 cm) was packed well in an airtight hardening box to provide limited oxygen conditions and pyrolyzed using the muffle furnace under 300, 500 and 700 °C (BC300, BC500 and BC700) according to the method described by Vithanage *et al.* with slight modifications.^33^ Briefly, the feedstock was heated to the desired temperature at a rate of 7 °C min^−1^ and held for 3 h. The hardening box was allowed to cool inside the furnace overnight to avoid air oxidation. The produced BC were washed with deionized (DI) water until washings were clear and oven dried at 60 °C for 12 h. Washed BC was ground and sieved to retain the 0.5–1 mm mesh fraction. Samples were kept in desiccators until use.

### 2.3. Proximate and ultimate analysis

Ash and moisture contents of BC were determined according to the American Society for Testing and Materials (ASTM) D2866-94 and D2867-17 methods respectively.^34,35^

The C, H, O and N compositions were used to determine polarity indexes. The H/C atomic ratios were used as an indication of aromaticity. Furthermore, O/C ratios and polarity indexes ((O + N)/C ratios) were calculated to evaluate polarities of the produced BCs. Thermogravimetric analyses were performed under 100 mL min^−1^ nitrogen flow in the temperature range of 30–800 °C. About ∼10 mg of accurately weighed samples were used in each experiment. Statistical analyses were performed using R Core Team (2013). R: a language and environment for statistical computing. R Foundation for Statistical Computing, Vienna, Austria.

### 2.4. Acid modifications of BC

Post modifications were carried out according to previously reported procedures with slight amendments.^20,36,37^ For the SM, a 100 mL portion of 10% H_2_SO_4_ was added to 10 g of BC and stirred for 1 h at 60 °C.^36^ The NM was performed by stirring 10 mL of 69% HNO_3_ with 10 g of BC for 3 h at 60 °C.^20^ For the HM, a 100 mL portion of 5 M HCl was added to 10 g of BC and stirred for 24 h at 50 °C.^37^ The modified BC was oven dried at 60 °C for 12 h after washing with DI until the washings became clear and neutral. The SM, NM and HM BCs will hereafter be abbreviated as SMBC, NMBC and HMBC respectively.

### 2.5. Surface morphology and functionality

To obtain SEM images, samples were coated on a carbon stub attached to a carbon tape and then was mounted onto a sample holder for SEM analysis. Surface functionality was evaluated by obtaining an ATR-FTIR spectra of the dry BC within a range of 4000–500 cm^−1^ with 4 cm^−1^ resolution through 64 scans.

The monochromatic X-ray source of the XPS analyzer was set to 1486.6 eV, corresponding to the Al K_α_ line, with a spot size of 400 μm^2^. Photoelectrons were collected from a takeoff angle of 90° relative to the overall sample's fractal particle surface. Measurements were done in the constant analyzer energy mode. The survey spectra were taken at a pass energy of 200 eV, while the high resolution core level spectra were taken at a 40 eV pass energy.

### 2.6. Measurements of EC, pH and PZC

Biochar pH and EC measurements were carried out according to the method described elsewhere with slight modifications.^33^ A 1 : 10 (w/v) BC: water suspension ratio was equilibrated at 100 rpm in a mechanical shaker at room temperature for 4 h prior to measurements. Nitrogen purged DI was used for experiments in order to eliminate dissolved CO_2_ and the data were corrected with respect to a simultaneous blank run carried out using DI water.

The PZC of BC was determined by the pH drift method described in Ferro-Garcia *et al.* Briefly, 60 mg samples of BC were shaken with 20 mL portions of 0.01 M NaCl solutions pre-adjusted to pH 2, 4, 6, 8, and 10 with 0.1 M HCl or NaOH for 24 h at 25 °C and the final pH was measured. Sodium chloride solutions were purged with N_2_ upon preparation to prevent the dissolution of CO_2_.

### 2.7. Surface acidity and basicity

Acidic and basic functional group distribution on the BC surface was determined by performing Boehm titrations. To evaluate surface acidity, an accurately weighed aliquot of BC (∼0.5000 g) was shaken either with 50 mL portions of 0.05 M NaHCO_3_, Na_2_CO_3_, or NaOH for 12 h. After equilibration, the suspension was filtered and titrated with 0.05 M HCl using the autotitrator. The Boehm titration assumes that NaHCO_3_ neutralizes stronger acids (mainly carboxylic), Na_2_CO_3_ neutralizes stronger and moderate acids (mainly low p*K*_a_ phenols and hydrolysis products of lactones), and NaOH neutralizes all acidic moieties including weak acids (mainly high p*K*_a_ phenols). Surface basicity was determined by shaking BC with 0.05 M HCl and subsequently titrating with 0.05 M NaOH in a similar manner. Biochar samples were pretreated with NaOH to remove solubilizable acidic species and humic substances, and afterwards with HCl to remove solubilizable basic species and to protonate O-SFGs as described by Tsechansky *et al.*^38^

### 2.8. Cation exchange capacity

The ammonium acetate compulsory displacement method described by Sumner and Miller was utilized for CEC determinations with slight modifications.^39^

Concisely, accurately weighed aliquots of BC (∼1.000 g) were shaken in pH adjusted sodium acetate (0.5 M solutions adjusted to pH = 3, 7 and 10 using HCl and NaOH) at 200 rpm for 1 h and filtered under vacuum using a Whatman 42 filter paper. The BC was washed five times with 30 mL portions of the same acetate solution to ensure the saturation with sodium ions. Biochar samples were then washed three times with 20 mL of ethanol to remove excess of sodium ions and the washings were discarded. Exchanged sodium ions were displaced with 30 mL of 0.5 M ammonium acetate five times and the collected washings were quantitatively diluted and measured using the AAS.

## 3. Results and discussion

### 3.1. Pyrolysis temperature selection

Biochar production entails the conversion of hemicellulose, cellulose and lignin fractions of the biomass into disordered recalcitrant organic carbon which has high stability and a life span of more than a century, depending on production conditions.^40–42^ The devolatilization and carbonization of hemicellulose and cellulose begins at a temperatures starting from 180–240 °C and 230–310 °C respectively. Decomposition of lignin starts from temperatures as low as 160 °C and continues slowly until up to 900 °C.^11^ The lower margin of BC production temperature by slow pyrolysis can be considered as ∼300 °C as only a limited decomposition of hemicellulose is observed at lesser temperatures.^43^

Aromatization reactions predominate at temperatures beyond 400 °C and the fraction of aromatic domains are rapidly increased at elevated temperatures.^44^ When the temperature exceeds 700 °C, the carbon structure becomes more ordered and graphitized increasing the size of aromatic domains.^45,46^ Turbostratic structures have been observed at temperatures as high as 800 °C and the structure becomes graphitic with order in the third dimension when the temperature exceeds 1500 °C, significantly reducing the stability and porosity of BC.^46^ Hence, BC for the presented study were prepared at the two borderline temperatures, 300 and 700 °C and at an average temperature of 500 °C.

### 3.2. Surface morphology and functionality

#### 3.2.1. Surface morphology

**Fig. 1:**
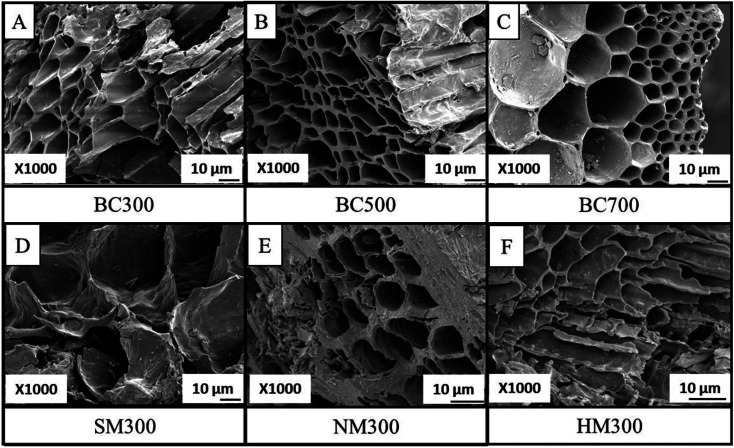
SEM images of raw BCs (A) BC300, (B) BC500 and (C) BC700, and modified BCs (D) SM300, (E) NM300 and (F) HM300.

The amorphous and heterogeneous structure of TWBCs were confirmed by SEM images. Variation of surface morphology with pyrolysis temperature is well documented in literature through SEM image based observations.^26,47–49^ It has been reported that the orderliness of the graphitized structure and the degree of aromaticity increased with the pyrolysis temperature due to the volatilization of oxygen and hydrogen from the low aromaticity lignocellulosic compounds and lignin causing the remaining carbon to form new aromatic bonds.^47^ Release of volatile compounds create a considerable internal pressure causing the coalescence of the smaller pores, forming more enlarged pores.^26,48,49^ Further, SEM images of BC produced under high temperatures have provided indirect evidence of the release of volatile compounds during pyrolysis. Obtained SEM images shown in Fig. 1A–C support the above phenomena. Even though pore wall destruction can be seen upon all three acid modifications, sulphuric acid treatment showed maximum effect due to the surplus water vapor released through the dehydration of sulphuric acid than the nitric and hydrochloric acids according to SEM images (Fig. 1D–F).^20^ Surface areas were increased upon increasing pyrolysis temperature and BC700 showed considerable increase upon acid modifications (Table S1, ESI†).

#### 3.2.2. FTIR analysis

**Fig. 2:**
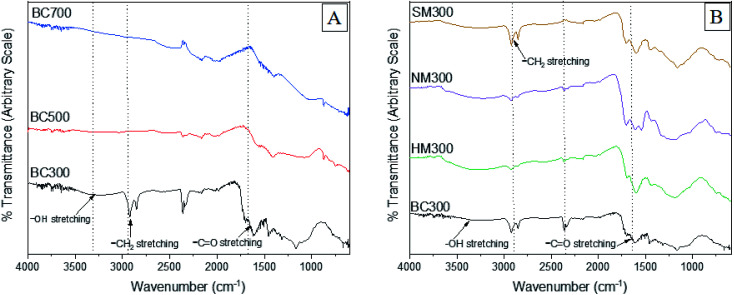
FTIR spectra of (A) raw BCs and (B) modified BCs.

The variation of FTIR spectra of TWBC with pyrolysis temperature and different acid modifications are shown in Fig. 2. The SFGs of BC are derived by the decomposition of organic components in feedstock such as lignin, cellulose and hemicellulose. Three major bands of FTIR spectra were identified at wavenumbers 3200–3500 cm^−1^ and 2820–2980 cm^−1^ corresponding to –OH stretching of alcohol/carboxylic and –CH_2_ stretching of polar groups respectively. The peak at 885–750 cm^−1^ is representative of out of plane isolated and substituted C–H in the aromatic structure and also of carbonates present. The XPS data (Section 3.2.3) provides validation for the presence of carbonates in the biochar structure. Overall intensity of FTIR spectra was decreased with increasing pyrolysis temperature. The diminution of the alcohol/carboxylic –OH stretching can be attributed to dehydration whereas the disappearance of –CH_2_ aliphatic bands indicated a decrease in polar functional groups.

An intense peak assigned to carbonyl stretching was observed around 1690–1720 cm^−1^ in NMBC and SMBC due to the introduction of carboxylic acids. A peak around 1200–1250 cm^−1^ in HMBC representing C–O stretching bond can be due to the formation of singly bonded C–O moieties in lactones and phenols. The appearance of a band at 874 cm^−1^ in BC500 and BC700 indicated the formation of larger aromatic domains.^32,49,50^ A similar observation was reported by Ahmed *et al.*^50^

#### 3.2.3. XPS analysis

Low resolution XPS spectra for BC300, BC500, BC700 and acid modified BC (SM, HM, NM) are given in ESI (Fig. S2†). The survey spectrum shows changes in the elemental composition upon acid treatment. Traces of Mg, Mn and Si are detected in BC precursors and Mg and Mn are seen to disappear with acid treatment. This could be due to the dissolution of Mg and Mn oxides upon acid treatment. However, oxides of Si are highly stable in these acids and not removed during acid modification.

**Fig. 3:**
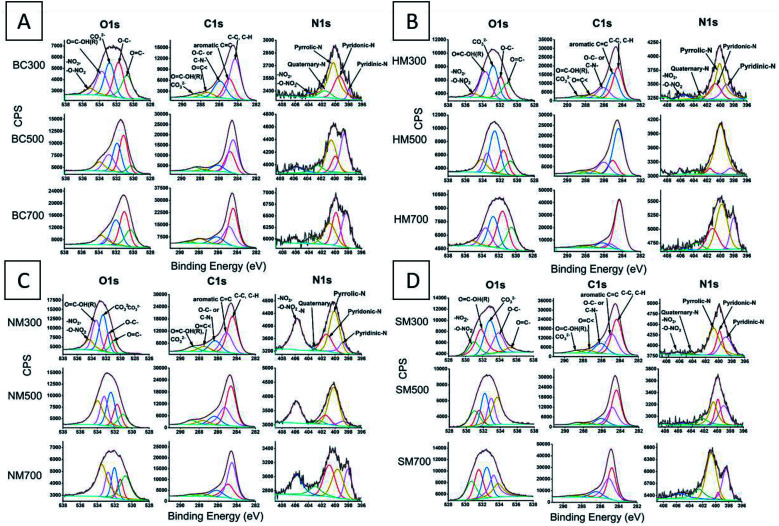
High resolution (HR) O1s, C1s and N1s XPS spectra for (A) raw BC, (B) HMBC, (C) NMBC and (D) SMBC.

The O1s high resolution envelope for BC300, BC500 and BC700 and BC after acid modification was resolved into five signals. The XPS spectra for raw and modified BC300, BC500 and BC700 are given in Fig. 3, while the associated data of the raw and modified BC are given in the ESI (Tables S3–S6†).

The highest energy contribution in 535.1–533.7 eV range can be attributed to–NO_2_, –O–NO_2_ functionalities. A binding energy (BE) range of 533.7–532.88 eV can be attributed to O=C–OH(R) functionalities. Carbonate (CO_3_^2−^) type functionalities occur at 532.8–531.99 eV BE range. O–C– and O=C– functionalities exist in 531.74–531.03 eV and 530.26–530.75 BE range respectively.^51^ When increasing the pyrolysis temperature from 300 °C to 500 °C, O=C–OH(R) atomic percentage have increased slightly. This could be due to an increase in carbonates with increasing pyrolysis temperature and partial overlapping of O=C–OH(R) with CO_3_^2−^ spectrum leading to a net increase in atomic percentages. But however, with further increase in temperature the atomic percentage drops drastically. This can be attributed to the loss in O=C–OH(R) functionalities because of decarboxylation at high temperatures.

The C1s high resolution envelop was curve resolved into four signals which are listed and assigned in Table S3† and Fig. 3 respectively. The highest energy contribution can be attributed to O=C–OH(R), CO_3_^2−^ in BE range of 289.21–288.53 eV. C=O/–C–N, and O–C– occurs in 287.89–287.28 eV and 286.13–284.92 eV (BE) range respectively. C–C and C–H co-occur in BE 284.46–284.28 eV range.^51^ Similar to O1s O=C–OH(R), CO_3_^2−^ atomic percentage increased with increasing temperature and dropped with further increment. Individual contribution from CO_3_^2−^ and O=C–OH(R) cannot be estimated as they co-exist. O–C– has been decreased with increasing carbonization temperature. Aromaticity of the char has been increased with the carbonization temperature.

The N1s high resolution envelop has been curve resolved into five peaks. The highest BE can be attributed to –NO_2_, –O–NO_2_ (496.26–494.08 eV BE). Quaternary-N occurs around BE 403.51–401.54 eV range. The pyrrolic-N, pyridonic-N and pyridinic-N occurs in the 400.81–400.29 eV, 399.91–399.37 eV and 398.62–397.96 eV range respectively.^52^ Quaternary-N shows no significant change with increasing pyrolysis temperature. However, the pyrrolic-N atomic percentage has been decreased with increasing pyrolysis temperature possibly due to deamination. Subsequently, the aromatic pyridonic-N and pyridinic-N has been increased which further suggests that the aromaticity of the BC has been increased with increasing pyrolysis temperature.

Upon nitration, the nitro and nitroxy atomic percentages (in O1s and N1s) have been increased in NM300, NM500 and NM700 compared to their precursor BCs; BC300, BC500 and BC700 respectively, and with increasing pyrolysis temperature they further increase (Fig. 3). This is due to the availability of aromatic rings to drive the nitration reaction. C1s O=C–OH(R), CO_3_^2−^ atomic percentages have been increased and this can be attributed to the increase in O=C–OH(R) moieties upon NM.

Hydrochloric acid modification resulted in low surface acidic functionalities (1.74–3.63% in O1s and 2.8–3.25% in C1s) compared to the precursor BCs. Also, the carbonate atomic percentages have been decreased compared to the precursor BCs, as HCl can titrate the carbonates of Mg, Ca and Na *etc.*

**Fig. 4:**
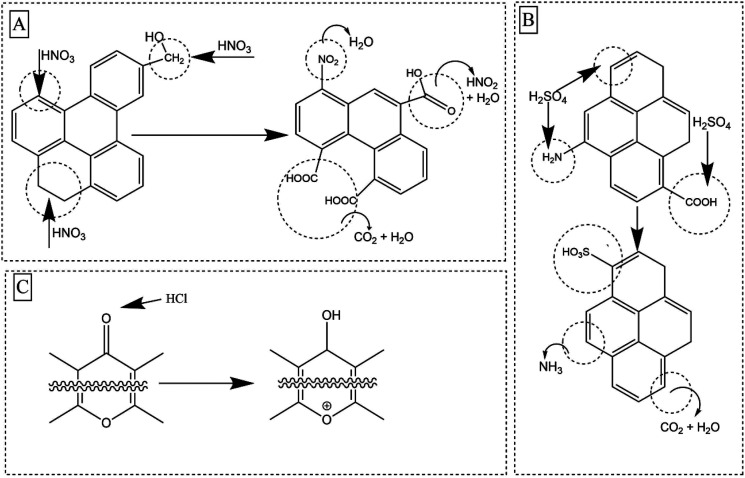
Proposed reaction mechanisms involved in (A) nitric, (B) sulfuric and (C) hydrochloric modification.

Sulfuric acid modification has failed to increase any carboxylic acidic functionalities compared to its precursor BC as, it can catalyze the decarboxylation of existing carboxylic groups.^24^ However, several new peaks appeared in S2p for the SM700. The S2p high resolution envelope was deconvoluted to two peaks S–H, C–S–C (166.3–163.3 eV) and –C–SO_2_–C (173.0–165.9 eV) (Fig. S7†). S2p only appeared in SM700. This could be due to high aromaticity present in SM700 and sulfonation being readily possible due to the high aromaticity. Presence of C–S–C peak suggests that the BC has been slightly cross linked with sulfur. Possible key reaction mechanisms upon the three acid modifications are shown in Fig. 4.

### 3.3. Surface acidity and basicity

**Fig. 5:**
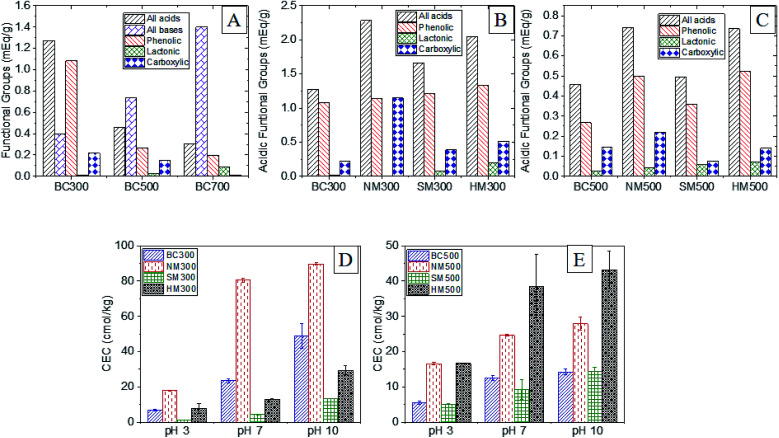
Surface Functional Groups (SFGs) comparison for (A) raw BCs, (B) modified 300BCs, (C) modified 500BCs and CEC variation under different pH values (D) modified 300BCs and (E) modified 500BCs.

A significant diminution of total surface acidity was observed with increasing pyrolysis temperature due to volatilization and degradation (Fig. 5A). Biochar produced at 500 °C contained 1.7 times more O-SFGs than BC700 and BC300 contained 2.7 times more O-SFGs than BC500. High p*K*_a_ phenols had the highest contribution to surface acidity in all three raw BCs (73–82%). Carboxylic moieties exhibited a 17% contribution to the total surface acidity of BC300 which was reduced to 8% and 5% in BC500 and BC700 respectively. The highest percentage of lactonic and low p*K*_a_ phenolic SFGs were observed in BC700 which was 21% while it was reduced to 5% in BC500 and to less than 1% in BC300. In contrast to surface acidity, increased surface basicities were observed as the pyrolysis temperature was increased (Fig. 5A). The production of basic moieties such as pyrones and chromens at elevated pyrolysis temperatures could be the reason for this observation.^53^

Boehm titrations were also performed for acid modified BC300 and BC500. Significant enhancements of total surface acidity were observed upon all three acid modifications (Fig. 5B and C). For both BC300 and BC500, the highest enrichment of total surface acidity was obtained upon NM (∼1.6–1.8 times) which was solely due to the introduction of carboxylic moieties. It has been reported that both the introduction of new O-SFGs *via* oxidative ring opening of aromatic rings and the oxidation of existing O-SFGs can occur during NM (Fig. 4A).^20,22^ An increment in lactonic groups was observed upon HM in both HM300 and HM500 as hydrochloric acid is capable of reducing O-SFGs.^24^ The reaction mechanism involved in the introduction of single-bonded O-SFGs is included in Fig. 4C. Among the three acid treatment methods, the lowest surface acidity enhancement was caused by the SM. The observed decrement in carboxylic moieties upon SM can be attributed to decarboxylation reactions (Fig. 4B).

The increment of specific surface area and the resultant exposure of unavailable SFGs caused by the demineralization effect during an acid treatment can also contribute to the enrichment of surface acidity.^54^ As the basic moieties on carbonaceous surfaces are already being neutralized by acid treatment, basicity evaluations of modified BC were not evaluated.

### 3.4. Proximate and ultimate analysis

#### 3.4.1. Yield, ash and moisture content

Maximum decomposition of biomass during pyrolysis occurs from ∼200–400 °C.^55^ The BC yield sharply decreased when the pyrolysis temperature was increased from 300 to 500 °C and only slightly decreased when it was further increased to 700 °C, indicating that the majority of the volatile fraction has been removed at lower temperatures (Table 1).

Proximate and ultimate analysis of raw and three acid modified TWBCs produced under three different pyrolysis temperatures^a^

**Table:** 

Sample	Proximate analysis					
	Yield (%)	Moisture (%)	Ash (%)	pH	pH_PZC_	EC (μS cm^−1^)
BC300	52.17 ± 1.28	6.33 ± 0.57	6.15 ± 0.22	7.16 ± 0.01	6.31	12 025.00 ± 0.07
BC500	32.58 ± 2.17	9.70 ± 0.60	11.40 ± 0.25	7.04 ± 0.02	9.16	2514.50 ± 0.07
BC700	28.17 ± 1.89	7.97 ± 0.04	9.26 ± 0.37	10.09 ± 0.05	7.51	1984.00 ± 1.41
NM300			2.80 ± 0.28	2.40 ± 0.01	4.67	1457.65 ± 0.70
NM500			3.10 ± 0.35	2.44 ± 0.00	4.96	1264.50 ± 0.70
NM700			2.50 ± 0.71	2.57 ± 0.01	5.55	1224.50 ± 1.41
SM300			4.00 ± 0.35	3.40 ± 0.01	5.35	1187.00 ± 1.41
SM500			3.50 ± 0.79	2.35 ± 0.04	5.45	1607.50 ± 0.07
SM700			6.50 ± 1.01	4.16 ± 0.00	6.95	663.00 ± 2.82
HM300			3.10 ± 0.18	2.55 ± 0.00	5.67	482.50 ± 0.70
HM500			3.80 ± 0.53	2.65 ± 0.00	5.60	418.00 ± 1.41
HM700			7.00 ± 0.83	2.49 ± 0.01	2.65	1191.00 ± 1.41

a NM represents nitric acid modification, whereas SM and HM represent sulfuric acid and hydrochloric acid modifications respectively.

**Table:** 

	Ultimate analysis						
	C (%)	H (%)	N (%)	O (%)	Molar H/C	Molar O/C	Molar (O + N)/C
Tea-waste	47.80	5.12	3.75	42.17	1.29	0.68	0.75
BC300	57.80	4.42	3.66	34.12	0.92	0.44	0.50
BC500	69.66	2.96	2.55	24.82	0.51	0.27	0.30
BC700	71.03	2.11	3.12	23.74	0.36	0.25	0.29
NM300	57.03	4.47	5.51	32.99	0.94	0.43	0.52
NM500	56.57	4.39	5.26	33.78	0.93	0.45	0.53
NM700	71.30	2.21	3.80	22.69	0.37	0.24	0.28
SM300	60.79	4.69	3.87	30.65	0.93	0.38	0.43
SM500	61.27	4.55	3.92	30.26	0.89	0.37	0.43
SM700	71.37	2.14	3.04	23.45	0.36	0.25	0.28
HM300	63.15	4.75	3.92	28.18	0.90	0.34	0.39
HM500	63.35	4.17	3.88	28.07	0.79	0.33	0.39
HM700	74.02	2.22	3.15	20.62	0.36	0.21	0.25

The ash content increased from 6.3% to 11.4% when the pyrolysis temperature was increased from 300 to 500 °C and slightly decreased to 9.3% when the temperature was further increased to 700 °C. Zhao *et al.* also reported a similar observation for apple tree branch BC.^55^ The possible reason for this observation may be the increased amount of inorganic materials in BC500 compared to BC300 and the partial devolatilization of produced inorganic constituents in BC700. Decreased ash contents were observed upon all three acid modifications suggesting the removal of trapped inorganic constituents (Table 1).

The C content of BC300 which was 58% was increased to 71% for BC700. In contrast, the oxygen content decreased from 34% to 24% when going from BC300 to BC700. These two facts indicated an increase in carbonization and a decrease in polarity when the temperature was increased.

#### 3.4.2. Elemental analysis

The molar O/C ratios and the (O + N)/C ratios were observed to decrease with increasing pyrolysis temperature stipulating that BC produced at higher temperatures were hydrophobic. This was as a result of the volatilization of polar functional groups. At higher pyrolysis temperatures, the molar H/C ratios were seen to decrease as well indicating that BC produced at elevated temperatures possessed a highly aromatic structure.^32^ A similar pattern was observed in acid modified BCs. Elemental compositions as well as H/C and (O + N)/C ratios of raw and modified BC are shown in Table 1.

#### 3.4.3. Thermo gravimetric analysis (TGA)

The TGA analysis was used to investigate changes in the thermal stability of the BC after the acid treatment process. Fig. S8† shows the TGA curves for raw BC, SMBC, HMBC and NMBC. The BC 300 possessed poor thermal stability, which could be due to the decarboxylation and, decomposition of remaining lignocellulose material.^56^ Biochar produced at 500 °C and BC700 are fairly stable in the TGA experiment temperature, as they were produced from high temperature pyrolysis. However, ∼15% of the weight loss can be attributed to the loss of volatile organic compounds (VOC) and decarboxylation of BC.^56^ Sulfuric acid modification decreased the stability of BC500 and BC700. Consequently, the stability of BC300 has been increased. This weight loss of SM500 and SM700 could be possibly due to loss of VOCs and de-sulfonation. The SM500 and SM700 may have more degree of sulfonation as their precursor BC's (BC500 and BC700) are more aromatic. Both HM and NM markedly decrease the graphitic and aromatic carbons in BC300 and BC500 and increases the oxygenated and nitrogenated functionalities.^24^ It leads to poor thermal stability of HM/NM 300 and 500. The aromaticity of BC700 is not susceptible or not changed significantly upon NM and HM which leads to higher thermal stability.

### 3.5. pH and electrical conductivity measurements

Organic matter, inorganic constituents and SFGs can be considered as the main factors affecting the pH of BC.^57^ Low temperature produced BC is commonly expected to have acidic pH as a result of the formation of O-SFGs and various other acidic organic substances while high temperature BC generally exhibits alkaline pH due to the higher rates of carbonization of organic matter and the removal of SFGs.^43^ However in this study, both BC300 and BC500 showed neutral pH while BC700 showed mildly basic pH (Table 1). It is interesting to note the insignificant difference in pH of BC300 and BC500 despite the variation of their ash content. Though BC300 contained a significantly higher amount of acidic O-SFGs as compared to BC500, their contribution to pH can be assumed to be negligible because the total surface acidity was mainly contributed to by weak, high p*K*_a_ phenols. It is also important to note the increment of basic SFGs and the decrement of acidic SFGs when the pyrolysis temperature was increased from 300 to 700 °C. Though the exact phenomenon for the pH variation with pyrolysis temperature is unclear, it can be concluded that the SFGs play a dominating role on the BC pH.

Dissolution of ash content in BC is a key factor contributing to the EC.^58^ Among the three raw BC, the highest EC was observed by BC500 (Table 1) and the high ash content justifies this observation. The release of the soluble organics such as humic substances in BC500 could also be a factor contributing to the elevated EC.

As a whole, ECs were reduced upon acid modifications (Table 1). The highest diminution in EC was observed in HMBC whereas the lowest diminution was observed in NMBC. The EC of NMBC300 was slightly higher than that of BC300 suggesting the contribution of a high amount of added carboxylic groups which are capable of providing protons to the medium.

### 3.6. Point of zero charge

For raw BC, only BC300 showed an acidic PZC and the highest PZC was observed in BC500. Low PZC of BC300 can be as a result of a high content of acidic O-SFGs and the highly basic PZC of BC500 can be due to the high ash content. The observed PZC values were decreased upon acid modifications (Table 1). This can be explained by the demineralization effect that lowers the ash content as well as the appearance of new acidic O-SFGs.

### 3.7. Cation exchange capacity

Cation exchange capacities of modified and raw BC produced at 300 and 500 °C were evaluated at pH of 3, 7 and 10. Significantly higher CECs were observed in BC300 when compared to BC500. As confirmed by Boehm titrations, BC300 contained a significantly higher amount of O-SFGs which can act as favorable binding sites.

Results also showed that the CEC for a given BC is increased with the pH of the medium (Fig. 5D and E). For raw and NMBC, sharper enhancements (3.5× and 4.5× respectively) of CEC were observed when the pH was increased from 3 to 7 whereas comparatively slighter enhancements were observed upon pH increment from 7 to 10 (2.1× and 1.1× respectively).

The contribution of carboxylic and lactonic groups can be a possible reason for this observation. Since the p*K*_a_ values of carboxylic and lactonic groups generally range from 2–4 and 4–7 respectively, it can be concluded that the majority of these O-SFGs can be in their deprotonated form when the pH reaches 7, acting as excellent binding sites for cations *via* electrostatic interactions. The slight increase in CEC when the pH was increased from 7 to 10 may be due to the deprotonation of phenolic groups which have lesser contribution to CEC.^59^

Acid modifications have generally increased the CEC. Introduction of binding sites, pore widening and opening of unavailable pores can be considered as the major reasons responsible for this observation.^3,20^ The enhancement of CEC at acidic pH can be mainly due to the two latter reasons as majority of O-SFGs are protonated. Highest enhancement of CEC in BC300 was achieved through NM due to the introduction of carboxylic groups. Though NM of BC500 showed a significant enhancement in CEC, the highest enhancement was observed *via* the HM. Since BC500 did not contain as much O-SFGs as BC300, pore opening and widening might have played a predominant role when determining CEC.

## 4. Statistical analysis

Due to small sample size (*n* = 3), a nonparametric ANOVA was conducted to find out whether there was a significant difference among the median value of a variable with respect to BC type. A Kruskal–Wallis test has been conducted. Significant differences were observed for the ash content of the raw, HMBC and SMBC with *p*-values of 0.027, 0.026, and 0.029 respectively. The difference in the ash content for NMBC was not statistically significant (*p*-value = 0.939). A significant linear relationship between the ash content and BC types for the modified BC was observed only for the HMBC.

Far from the normal BC types tested, for EC, the three different acid modified BC types showed a significant difference amongst them (*p*-values < 0.05) whereas for the normal BC, the difference was not significant (*p* value = 0.055).

There was a significant difference observed for the basic, acidic, lactonic and carboxylic groups present amongst the three types of normal BC (*p* value = 0.027). The median of phenolic groups however did not differ significantly (*p*-value = 0.051). For the two BC types which were subjected to acid modifications, the acidic, phenolic, lactonic and carboxylic moieties present differed significantly amongst them (*p*-value < 0.05).

The CEC was observed to be different for BC300 and BC500 at pH 3, 7 and 10. Considering the same BC type at two different pH values, the values obtained were again different. The same pattern in results were got for the modified BCs as well although at pH 10, SM300 and SM500 showed no significant difference in CEC (*p*-value = 0.077). Overall, there exists a significant difference in ash content, EC, acidic functional groups and CEC between the raw and modified BC types.

## 5. Conclusions

Clear evidence exist to support the influence of acid modifications on physicochemical properties of the TWBC. Increasing pyrolysis temperature affects the surface acidity and the CEC as it can result in the decrement of acidic functionalities on the surface of the BC. The surface acidity for the raw BC samples were mainly as a contribution of phenolic groups. Incorporation of acid modifications yielded in elevated acidity for all the BC types tested. Far from the other modified samples which were in concordance with the raw BC showing insignificant variations in phenolic moieties, there was significant evidence that an enhancement in carboxylic groups contributed to the surface acidity upon NM. The SM, however, did not result in the formation of any carboxylic acid groups to a significant level but advanced the decarboxylation of the existing functionalities. The acid modified BC samples had low PZC values and showed a decrease in the ash content as a result of the demineralization and the introduction of acidic functionalities. The acidic PZC value of the BC becomes advantageous as it allows for the sorption of cationic species under mildly acidic conditions through electrostatic interactions. Oxygenated surface functional groups present on the BC surface have a notable contribution to CEC as well, especially at high pH. At basic pH, the functional moieties are deprotonated and surface complexation promoted. For the BC300, the highest CEC was observed when it was subjected to NM and for the BC500, the highest value was observed in HM.

When the pyrolysis temperature was increased, the aromaticity (H/C) and polarity index (O + N)/C decreased indicating enhanced aromatization and hydrophobicity of the BC that resulted at elevated temperatures. The XPS and FTIR results obtained for the raw and modified samples were in close agreement and it can be seen that the nitro and nitroxy atomic percentages have increased for the NMBC produced at higher temperatures as the nitration reaction becomes more favored. The BC produced at high temperature showed high thermal stability as confirmed from the TGA experiments.

The NM on TWBC can be used for agricultural experiments because of its high CEC to amend the nutrient leaching effect in soils. The SEM images of the SMBC show pore destruction which makes the retention of bulky adsorbents possible. However, to get an insight about these effects upon application of modified BC, it is necessary to conduct extensive field experimentation.

## Conflicts of interest

There are no conflicts of interest to declare.

## Supplementary Material

RA-009-C9RA02729G-s001
