# Supplementary material for: The influence of three acid modifications on the physicochemical characteristics of tea-waste biochar pyrolyzed at different temperatures: a comparative study
Source: RSC Adv. 2019 Jun 4;9(31):17612–22. doi: 10.1039/c9ra02729g (PMC9064594; doi:10.1039/c9ra02729g)
Supplement: RA-009-C9RA02729G-s001 [file RA-009-C9RA02729G-s001.pdf]

Electronic Supplementary Material (ESI) for RSC Advances.
This journal is © The Royal Society of Chemistry 2019

Supplementary Information

The influence of three acid modifications on the physicochemical characteristics of tea-waste biochar pyrolyzed at different temperatures: a comparative study

Chathuri Peiris[1,2], Oshani Nayanathara[1], Chanaka M. Navarathna[3], Yohan Jayawardhana[4], Samadhi Nawalage[1], Griffin Burk[3], Akila G. Karunanayake[5], Sunith B. Madduri[6], Meththika Vithanage[7], M. N. Kaumal[2], Todd E. Mlsna[3], El Barbary Hassan[6], Sachith Abeysundara[8], Felio Ferez[9] and Sameera R. Gunatilake[1*]

[1]College of Chemical Sciences, Institute of Chemistry Ceylon, Rajagiriya, CO 10107, Sri Lanka

[2]Department of Chemistry, University of Colombo, CO 00300, Sri Lanka

[3]Department of Chemistry, Mississippi State University, MS 39762, USA

[4]National Institute of Fundamental Studies, Hantana, KY 20022, Sri Lanka

[5]Biochar Supreme LLC, Everson, WA 98247, USA

[6]Department of Sustainable Bioproducts, Mississippi State University, Mississippi State, MS 39762, USA

[7]Ecosphere Resilience Research Center, Faculty of Applied Sciences, University of Sri Jayewardenepura, Nugegoda, CO 10250, Sri Lanka

[8]Department of Statistics and Computer Science, University of Peradeniya, Peradeniya, KY 20400, Sri Lanka

[9]Material Science Lab, Integrated Microscopy Center, University of Memphis, Memphis, TN 38152, USA

Table S1: Surface area, Pore volume and diameter of tea waste Biochar (TWBC)

Biochar Type	Surface Area (m^2/g)	Pore Volume (cm^3/g)	Diameter (Å)
BC300	0.2038	0.000088	17.1877
BC500	0.5755	0.000200	13.8684
BC700	8.1053	0.002259	11.1459
NM300	0.2265	0.000069	12.1496
NM500	0.3362	0.000074	8.8366
NM700	216.3321	0.070649	13.0631
SM300	0.2032	0.000090	7.6925
SM500	0.1317	0.000025	7.5536
SM700	59.6797	0.017993	12.0599
HM300	0.0166	0.000180	11.5823
HM500	0.2138	0.000068	12.7930
HM700	110.7571	0.033150	12.0317

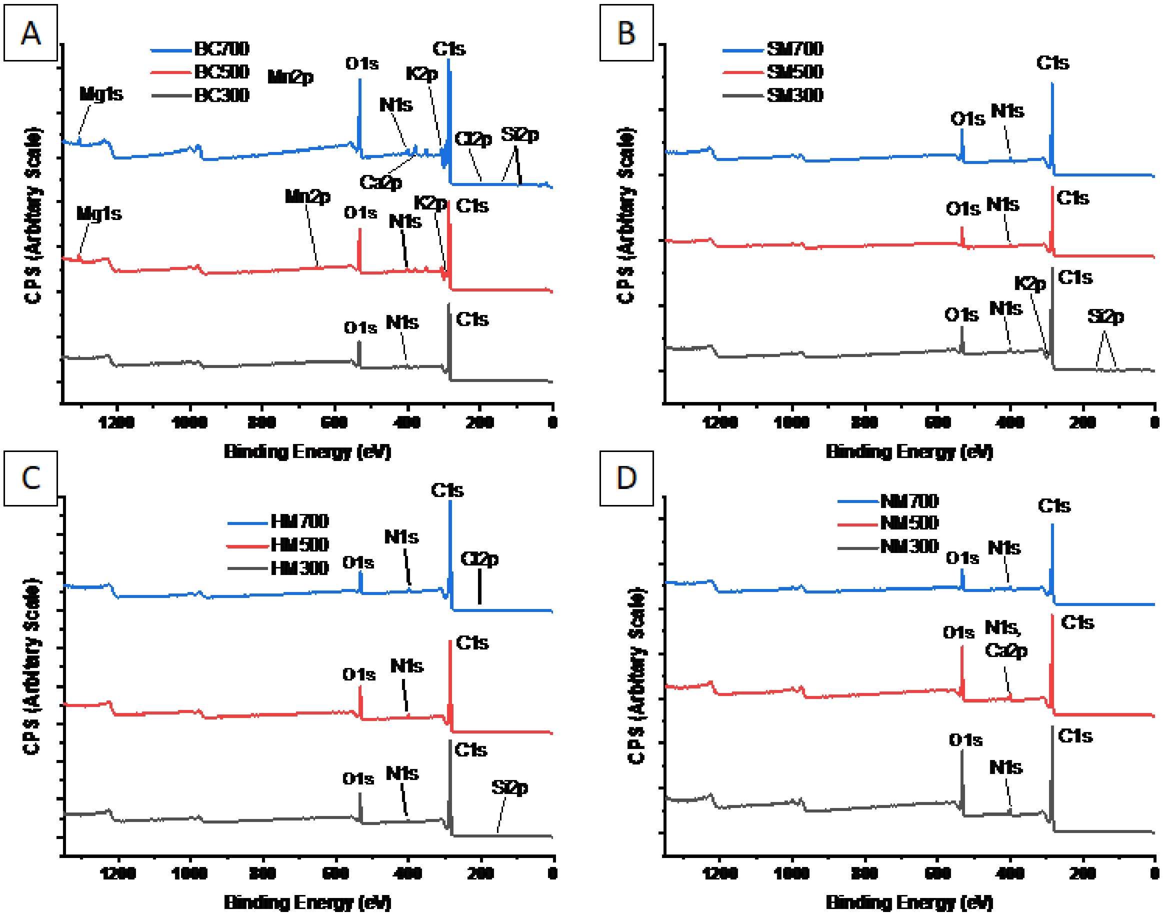

Figure S2: Low Resolution survey XPS Spectra for (A) Raw BC, (B) NMBC, (C) SMBC and (D)HMBC

Table S3: High resolution XPS data for BC300, BC500 and BC700

		BC300			BC500			BC700		
		BE(eV)	FWHM(eV)	Atomic %	BE(eV)	FWHM(eV)	Atomic %	BE(eV)	FWHM(eV)	Atomic %
O1s	$-NO_2$, $-O-NO_2$	535.1	1.47	1.02	533.99	1.4	2.3	533.7	1.5	3.24
	O=C-OH(R)	533.72	1.5	3.3	532.88	1.5	3.91	533	0.92	1.12
	CO_3^{2-}	532.8	1.35	4.12	531.92	1.17	5.24	531.99	1.43	6.59
	-O-C-	531.74	1.34	4.28	531.16	1.17	6.83	531.03	1.26	7.84
	O=C-	530.75	1.45	3.37	530.26	1.06	1.46	530.33	1.44	4.6
C1s	O=C-OH(R), CO_3^{2-}	288.58	1.48	3.89	288.53	1.48	6.05	289.21	1.43	1.88
	C=O	287.28	1.5	5.11	287.63	1.21	2.04	287.89	1.5	5.73
	-O-C and -C-N	285.91	1.5	16.93	286.12	1.5	8.31	286.13	1.5	8.62
	Aromatic C=C	284.92	1.31	17.45	284.78	1.25	23.31	284.81	1.33	21.55
	C-C, C-H	284.28	1.35	37.67	284.46	1.21	37.32	284.4	1.11	35.71
N1s	$-NO_2$, $-O-NO_2$	404.08	0.49	0.04	405.71	2.45	0.33	406.26	1.24	0.18
	Quaternary-N	401.54	2	0.25	402.48	1.8	0.35	403.51	1.72	0.22
	Pyrrolic-N	400.29	1.84	1.61	400.51	1.78	1.08	400.81	2.15	0.86
	Pyridonic-N	399.37	1.94	0.83	399.91	1.31	0.43	399.85	1.41	0.87
	Pyridinic-N	397.96	0.5	0.12	398.62	1.35	1.04	398.3	1.59	1

Table S4: High resolution XPS data for NM300, NM500 and NM700

		NM300			NM500			NM700		
		BE(eV)	FWHM(eV)	Atomic %	BE(eV)	FWHM(eV)	Atomic %	BE(eV)	FWHM(eV)	Atomic %
O1s	$-NO_2$, $-O-NO_2$	534.34	1.48	2.8	533.98	1.48	5.5	533.49	1.52	5.05
	O=C-OH(R)	533.36	1.31	6.07	533.21	1.04	3.91	532.74	0.85	1.69
	CO_3^{2-}	532.41	1.33	7.48	532.44	1.12	4.96	532.02	0.83	2.01
	-O-C-	531.56	1.04	3.15	531.71	1.02	3.06	531.34	0.76	1.24
	O=C-	530.89	1.25	2.63	531.04	1.3	2.42	530.75	1.44	2.91
C1s	O=C-OH(R), CO_3^{2-}	288.71	1.45	5.01	288.84	1.44	4.72	288.89	1.48	3.26
	C=O	287.47	1.5	5.11	287.6	1.5	4.54	287.62	1.5	3.61
	O-C or C-N	286.3	1.45	11.01	286.37	1.5	9.3	286.14	1.5	10.17
	Aromatic C=C	285.07	1.41	18.52	285.27	1.46	20.98	284.89	1.44	21.66
	C-C, C-H	284.44	1.28	32.82	284.6	1.22	35.98	284.5	1.1	43.7
N1s	$-NO_2$, $-O-NO_2$	405.71	1.84	1.82	405.85	1.83	1.35	405.76	1.95	0.93
	Quaternary-N	403.09	0.66	0.05	402.44	1.5	0.16	403	1.98	0.48
	Pyrrolic-N	401.28	2	0.96	401.45	1.67	0.49	400.86	2	1.33
	Pyridonic-N	400.09	1.78	2.08	400.2	1.87	2.31	399.62	2	1.2
	Pyridinic-N	398.82	1.33	0.5	398.77	1.7	0.31	398.06	1.23	0.77

Table S5: High resolution XPS data for HM300, HM500 and HM700

		HM300			HM500			HM700		
		BE(eV)	FWHM(eV)	Atomic %	BE(eV)	FWHM(eV)	Atomic %	BE(eV)	FWHM(eV)	Atomic %
O1s	$-NO_2$, $-O-NO_2$	535.07	1.47	0.55	534.12	1.4	2.34	535.09	1.5	0.59
	O=C-OH(R)	533.7	1.5	3.63	533.46	1.05	2.06	533.55	1.5	1.74
	CO_3^{2-}	532.78	1.33	4.05	532.59	1.39	5.87	532.66	1.42	2.73
	-O-C-	531.93	1.12	2.35	531.59	1.17	2.85	531.56	1.5	3.52
	O=C-	531.14	1.46	2.57	530.77	1.44	2.11	530.49	1.44	2.16
C1s	O=C-OH(R), CO_3^{2-}	288.85	1.45	2.8	288.72	1.46	3.24	288.8	1.49	3.25
	C=O	287.4	1.5	3.77	287.48	1.5	3.54	287.63	1.46	3.09
	O-C or C-N	286.05	1.5	13.7	286.02	1.5	13.56	286.14	1.5	8.6
	Aromatic C=C	285	1.33	29.61	285.04	1.19	12.72	285.36	1.23	6.23
	C-C, C-H	284.49	1.21	34.36	284.42	1.33	48.73	284.31	1.15	65.01
N1s	$-NO_2$, $-O-NO_2$	405.59	2.02	0.23	406.09	0.58	0.11	401.2	2	0.67
	Quaternary-N	402.96	0.51	0.05	404.03	2	0.14	406.5	2.02	0.16
	Pyrrolic-N	400.76	2	0.55	401.57	1.06	0.2	403.59	2	0.23
	Pyridonic-N	400.1	1.51	1.27	399.98	1.93	2.31	397.99	1.21	0.59
	Pyridinic-N	399.06	1.68	0.5	398.46	1.48	0.22	399.78	2	1.42

Table S6: High resolution XPS data for SM300, SM500 and SM700

		SM300			SM500			SM700		
		BE(eV)	FWHM(eV)	Atomic %	BE(eV)	FWHM(eV)	Atomic %	BE(eV)	FWHM(eV)	Atomic %
O1s	$-NO_2$, $-O-NO_2$	535.3	1.49	0.85	533.63	1.4	4.21	533.87	1.47	2.14
	O=C-OH(R)	533.87	1.5	2.85	532.93	0.98	2.32	533.33	1.2	2.98
	CO_3^{2-}	532.86	1.5	4.81	532.17	1.04	2.99	532.53	1.18	3.14
	-O-C-	531.81	1.46	4.01	531.49	1	1.42	531.59	1.19	3.06
	O=C-	531.02	1.44	2.02	530.98	1.43	2.09	530.72	1.42	2.4
C1s	O=C-OH(R), CO_3^{2-}	288.77	1.46	3.59	288.71	1.35	3.56	289.24	1.45	2.28
	C=O	287.47	1.5	3.77	287.47	1.5	2.78	287.83	1.5	3.26
	O-C or C-N	286.06	1.5	12.52	286.08	1.5	9.96	286.42	1.5	8.97
	Aromatic C=C	284.88	1.46	24.45	284.85	1.24	24.46	285.04	1.44	32.33
	C-C, C-H	284.34	1.28	38.39	284.43	1.16	43.83	284.74	1.03	34.78
N1s	$-NO_2$, $-O-NO_2$	406.42	2.5	0.16	405.65	2.54	0.13	405.52	2.54	0.35
	Quaternary-N	404.31	0.62	0.02	402.22	2.05	0.33	402.58	2.05	0.52
	Pyrrolic-N	400.75	1.97	1.25	400.58	1.39	0.71	400.69	1.83	1.63
	Pyridonic-N	399.87	1.12	0.58	399.89	1.02	0.53	399.63	0.55	0.11
	Pyridinic-N	398.89	2	0.73	399	1.84	0.68	398.42	1.31	0.86
S2p	$C-SO_2-C$							168.74	1.81	0.97
	S-H, C-S-C							164.05	1.33	0.23

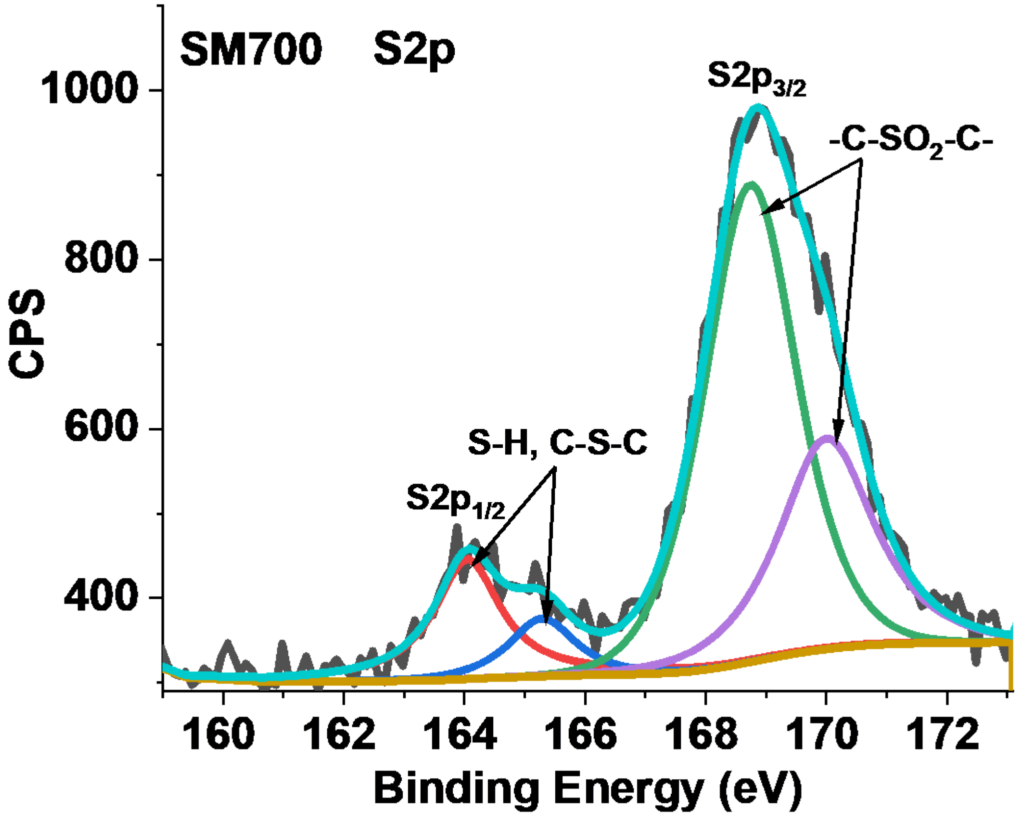

Figure S7: Resolved and assigned S2p HR-XPS spectra for SM700 (CPS – Counts per second).

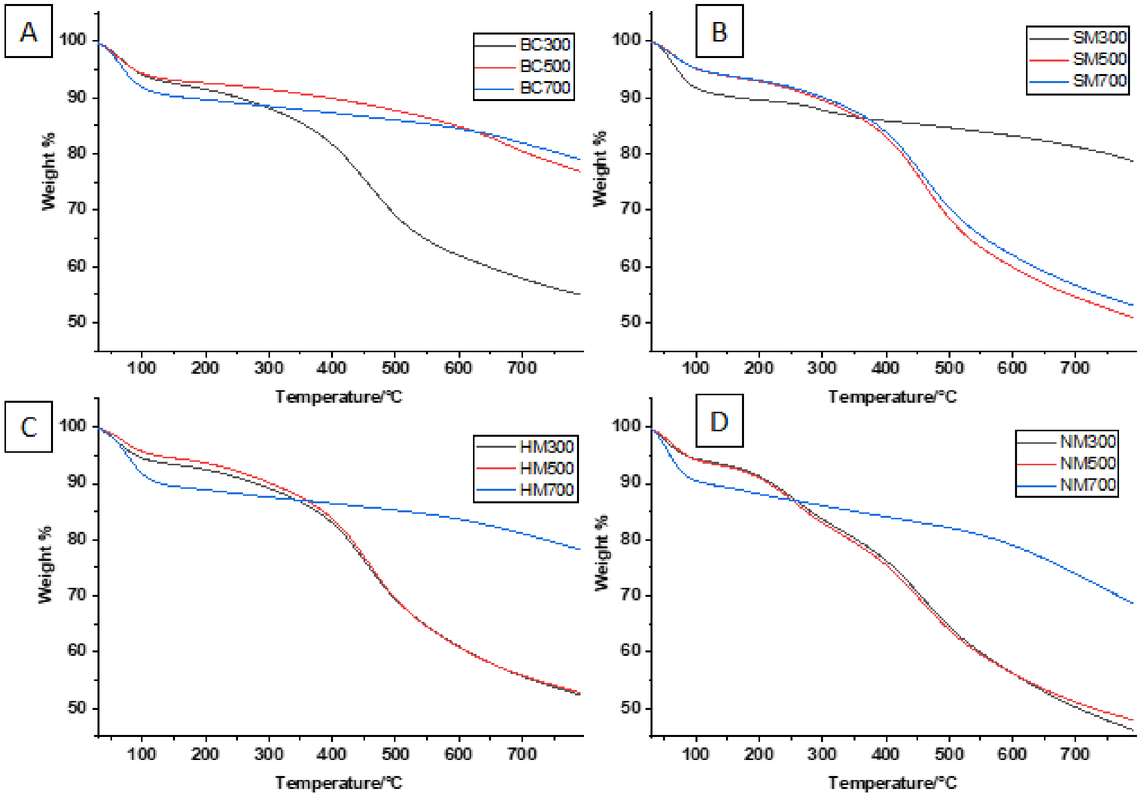

Figure S8: Low Resolution survey TGA Graphs for (A) Raw BC, (B) SMBC, (C) HMBC and (D)NMBC
